# The effects of social interactions on momentary stress and mood during COVID‐19 lockdowns

**DOI:** 10.1111/bjhp.12626

**Published:** 2022-10-17

**Authors:** Paul A. G. Forbes, Ekaterina Pronizius, Anja C. Feneberg, Urs M. Nater, Giulio Piperno, Giorgia Silani, Ana Stijovic, Claus Lamm

**Affiliations:** ^1^ Department of Cognition, Emotion, and Methods in Psychology, Faculty of Psychology University of Vienna Vienna Austria; ^2^ Department of Clinical and Health Psychology, Faculty of Psychology University of Vienna Vienna Austria; ^3^ University of Vienna Research Platform “The Stress of Life – Processes and Mechanisms underlying Everyday Life Stress” (SOLE), Faculty of Psychology University of Vienna Vienna Austria

**Keywords:** COVID‐19, ecological momentary assessment, mood, social interactions, stress

## Abstract

**Objective:**

Social interactions are vital for our well‐being, particularly during times of stress. However, previous studies linking social interactions to psychological outcomes during the COVID‐19 pandemic have largely been retrospective and/or cross‐sectional. Thus, we tested four preregistered hypotheses (H1–H4) concerning the *real‐time* effect of social interactions on momentary changes in stress and mood during two COVID‐19 lockdowns.

**Design:**

We used an ecological momentary assessment approach in 732 participants in spring 2020 (burst 1) and in a subsample of these participants (*n* = 281) during a further lockdown in autumn/winter 2020 (burst 2).

**Methods:**

Participants reported their stress and mood in a smartphone app five times per day for 7 days and indicated the nature and frequency of their recent social interactions.

**Results:**

Social interactions (H1) and their frequency (H2) improved momentary affect (e.g., social interactions increased mood valence: estimate = 2.605, *p* < .001 for burst 1). This was particularly the case for face‐to‐face interactions which, compared with other types of interactions, reduced momentary stress (e.g., estimate = −2.285, *p* < .001 for burst 1) and boosted mood (e.g., estimate = 1.759, *p* < .001 for burst 1) across both lockdowns, even when controlling for the pleasantness of the interaction and the closeness of the interaction partner (H3). We also show that individual differences in people's responsiveness to different social rewards modulated the impact of social interactions on momentary mood (H4).

**Conclusions:**

This study extends findings from cross‐sectional and retrospective studies by highlighting the real‐time affective benefits of social interactions during COVID‐19 lockdown. The results have important implications for the (self‐) management of stress and mood during psychologically demanding periods.

**Statement of contribution**

**What is already known on this subject?**

- Cross‐sectional studies showed the importance of social interactions during COVID‐19 lockdown.
- People who were generally more connected reported lower distress and better mood.

**What does this study add?**

- We show the *real‐time*, *within‐participant* benefits of social interactions during lockdown.
- Face‐to‐face interactions were especially important for improving momentary mood.
- Responses to different social rewards modulated the impact of social interactions.

## INTRODUCTION

The coronavirus disease 2019 (COVID‐19) spread exponentially across the world in 2020 resulting in a global pandemic. This created significant social and economic disruption resulting in uncertainty and uncontrollability (Ramiz et al., 2021)—which are the defining features of a stressor (Dickerson & Kemeny, 2004). Various forms of ‘lockdown’ (i.e., rules and regulations that confined citizens to their homes) were implemented by governments across the world to try and reduce the spread of the virus. These lockdowns, while varying in their severity, length and frequency, greatly limited people's ability to interact with others. For example, in Austria in March 2020, and again in November 2020, non‐essential businesses, including bars, restaurants, sport facilities and clubs, were closed and schools, universities and many places of work switched to remote learning and working. People were only allowed to leave their homes for a limited number of reasons, for example, to buy food or medicine, for work, or to exercise (Bundesministerium für Soziales, Gesundheit, Pflege und Konsumentenschutz, 2020). Similar restrictions on people's social lives were in place in most European countries, including Italy and Germany.

Social interactions are vital for our physical and mental well‐being (Sun, Harris, & Vazrie, 2020), particularly during times of stress (Lippold et al., 2016; Sachser et al., 1998). Multiple survey studies during the COVID‐19 pandemic demonstrated that different measures of our social relationships, such as social support (Szkody et al., 2021) and social network size (Nitschke et al., 2021), were associated with reduced distress during this psychologically demanding period. In one of the largest studies (*n* = 71,117), Sommerlad and colleagues (Sommerlad et al., 2021) collected weekly data from March to August 2020 in the United Kingdom. They found that both daily face‐to‐face contact and daily phone/video contact were linked to reduced depressive symptoms, but in‐person (i.e., face‐to‐face) contact appeared to be particularly beneficial (Subrahmanyam et al., 2020).

These surveys have proved important in identifying individuals who may be most at risk from the negative psychological effects of lockdown. For example, Sommerlad and colleagues (Sommerlad et al., 2021) showed that the relationship between social contact and depression was strongest in participants scoring higher on empathic concern and those who reported being more socially active prior to the pandemic. Yet, these studies typically ask participants to report *retrospectively* how they *generally* felt and behaved in the past week or month. So they cannot inform us about the *real‐time* effects of individual social interactions on a person's momentary stress and mood (Liu et al., 2019). Thus, in the current study, we aimed to build upon and complement these retrospective studies by using an ecological momentary assessment (EMA) approach (Shiffman et al., 2008). Participants entered their momentary stress and mood, as well as information about their recent social interactions, into a smartphone app five times per day for a seven‐day period during COVID‐19 lockdown in April and May 2020 (burst 1). To test the temporal robustness of our effects and the extent to which the findings generalized across separate lockdowns, a subsample of the participants residing in Austria from burst 1 took part in the study again in November and December 2020 following a further lockdown (burst 2).

We had four preregistered hypotheses (H1–H4) concerning the effect of social interaction on momentary stress and mood during lockdown (see https://osf.io/gsvdf ‘[4] Stress, mood, and social interactions’). Firstly, based on previous EMA studies (Liu et al., 2019), we predicted that social interactions (H1) and their frequency (H2) would be related to reduced momentary stress and improved mood during this psychologically demanding period. Moreover, given the unique context of lockdown during which people were largely confined to their homes, this changed both *how* people interacted and *who* they could interact with (Sun, Folarin, et al., 2020). Thus, we aimed to determine how the nature of social interactions predicted changes in momentary stress and mood. Specifically, we predicted that the medium (face‐to‐face vs. not face‐to‐face, e.g., via audio or text) and valence (i.e., pleasantness) of the interaction, and who it was with (i.e., the closeness of the interaction partner, e.g., family, stranger) would modulate the effect of social interactions on momentary stress and mood (H3). Finally, we aimed to establish whether certain individuals benefitted more from social interactions in terms of their momentary affective states. Individual differences in the ability to empathize with others and how socially active people were before the pandemic have been shown to modulate the effect of social relationships on depression (Sommerlad et al., 2021). We aimed to extend this work by establishing whether the potential stress‐buffering and mood‐boosting effects of social interactions were strongest in people who generally enjoy socializing in groups and having kind, reciprocal relationships (H4), as measured, respectively, by the Sociability subscale and the Prosocial Interactions subscale from the Social Reward Questionnaire (Foulkes et al., 2014).

## METHOD

### Participants

We used the *ema*.*powercurve* function from the R package *EMAtools* (Kleiman, 2017) to estimate the sample size given five entries per day across 7 days. To detect a small effect size with a mean data completion rate of 75%, we required 350 participants assuming 80% power (see https://osf.io/gsvdf). For burst 1, we recruited a convenience sample of 951 participants who downloaded the app and provided at least one data entry. These participants were recruited through various channels including an existing participant database, online advertisements and social media. 732 of these participants were included in the final sample (516 [70.49%] female, mean [*SD*] age = 31.66 [11.73]): 480 were in Austria during the seven‐day EMA period, 226 in Italy and 26 in Germany (see Table 1). Austria and Italy were the main countries of interest for burst 1 as this is where we predominantly advertised the study. However, as similar lockdown restrictions were in place in Germany during burst 1, we also included participants residing there. All participants in the final sample were 18 years old or older, provided data in at least 50% of the data entries (to ensure a mean completion rate of 75% across all participants, as per the power calculation) and completed the online questionnaire at the end of the study period. These criteria were determined a priori in the preregistration. Upon completion of the study, participants received 20€, which they could keep or donate to charity, and were also entered into a draw where they had the chance to win an additional 100€ voucher. For burst 2, we collected data during a further lockdown in Austria so recontacted participants residing in Austria from burst 1. This resulted in 357 participants from burst 1 providing at least one data entry for burst 2. The final sample included 281 (221 [78.65%] female, mean [*SD*] age = 34.18 [13.16]) participants (see Table 1), as for burst 1 we excluded participants who were younger than 18, provided data in less than 50% of the data entries or failed to complete the online questionnaire at the end of the study period. The study was approved by the local Ethics Committee (reference number 00553), and all participants provided informed consent online prior to taking part in the study.

**TABLE 1:** Sample characteristics for burst 1 and burst 2

	Burst 1	Burst 2	*p*
*N*	732	281	–
Country of residence	Austria = 480; Italy = 226; Germany = 26	Austria	–
Age	*M* = 31.66 (*SD* = 11.73) Range = 18–80	*M* = 34.18 (*SD* = 13.16) Range = 18–77	.005**
Gender	Female = 516 (70.49%); Male = 216	Female = 221 (78.65%); Male = 60	.009**
Completion rate	*M* = 78.63% (*SD* = 12.34) Range = 51.43–100	*M* = 76.58% (*SD* = 12.72) Range = 51.43–100	.021*
% social interactions	*M* = 72.63% (*SD* = 22.50) Range = 0–100	*M* = 70.31% (*SD* = 23.21) Range = 3.85–100	.153
% in person	*M* = 64.01% (*SD* = 29.04) Range = 0–100	*M* = 68.57% (*SD* = 26.00) Range = 0–100	.016*
Closeness	*M* = 79.40 (*SD* = 14.63) Range = 9.67–100	*M* = 73.75 (*SD* = 15.92) Range = 14.37–100	<.001***
Pleasantness	*M* = 74.29 (*SD* = 12.34) Range = 3–100	*M* = 73.74 (*SD* = 12.49) Range = 37.92–98.33	.526
Sociability	*M* = 5.11 (*SD* = 1.33) Range = 1–7	*M* = 4.72 (*SD* = 1.33) Range = 1–7	<.001***
Prosocial interaction	*M* = 6.51 (*SD* = 0.517) Range = 1–7	*M* = 6.47 (*SD* = 0.521) Range = 4–7	.174

*Note*: *Completion rate* refers to the proportion of data entries in which participants provided data. *Social interaction* refers to the proportion of completed data entries with a social interaction; *in person* refers to the proportion of completed data entries with a face‐to‐face interaction; *closeness* refers to the closeness of the social interaction partner in the most recent interaction; *pleasantness* refers to the pleasantness of the most recent social interaction. *Sociability* and *Prosocial Interactions* refer to the subscales from the SRQ (Foulkes et al., 2014). *p < .05; **p < .01; ***p < .001

### Procedure

For burst 1, data collection took place in April and May 2020 (Austria and Germany: 16 March–1 May; Italy: 9 March–8 May) when there were similar lockdown restrictions in place across Austria, Italy and Germany. For burst 2, data collection took place between 22 November and 8 December following the implementation of another lockdown on 17 November in Austria with similar restrictions as during burst 1 (see *Overview of lockdown restrictions during burst 1 and burst 2* in Supporting Information S1). Participants received a download link for an app, *movisensXS* (movisens GmbH, Karlsruhe, Germany), which allowed them to download the study in either German or Italian onto their personal smartphones. The app was programmed for Android so only those with Android devices could take part. The EMA period started the next day and lasted seven consecutive days. At the end of the EMA period, participants received a link to complete an online survey (SoSci Survey GmbH, Munich, Germany) in which they completed a battery of questionnaires, including the Social Reward Questionnaire (Foulkes et al., 2014). The study was part of a larger project registered on the Open Science Framework (for all items, see https://osf.io/rzqn6).

### Ecological momentary assessment

The EMA prompts occurred randomly between 10:00–11:00, 11:00–14:00, 14:00–17:00 and 17:00–20:00, with at least 90 minutes or more between prompts. The fifth and final data entry of each day was self‐initiated before participants went to bed. In burst 1, participants provided data on 78.6% of the prompts so on approximately 28 out of the 35 data entries. Similarly, for burst 2, participants provided data on 76.58% of the prompts (see Table 1). For each data entry, participants entered how stressed (‘At the moment, I feel stressed’) they felt on a visual analogue scale from 0 (‘not at all’) to 100 (‘very much’). Single‐item stress measures have been shown to have good validity (Lesage et al., 2012), which is why for reasons of practicality and measurement efficiency it was the ideal measure for this EMA study. Additionally, participants completed six bipolar items on a visual analogue scale, which loaded onto three dimensions of mood: valence (unwell‐well; dissatisfied‐satisfied), calmness (tense‐relaxed; restless‐calm) and energetic arousal (weak‐energetic; tired‐awake; items taken from [Wilhelm & Schoebi, 2007]).

During each data entry, participants were also asked, ‘Have you had an uninterrupted social exchange of more than 2 minutes since the last entry/since getting up?’ to which they could answer no or yes (and specify a number up to 10 from a drop‐down menu, or enter ‘more than 10’). Participants were informed, in either German or Italian, that:

An uninterrupted social exchange is any continuous interaction between you and at least one other person which lasted for more than 2 minutes. For example, this could include a conversation in real life/in person, a phone call or video call, and text‐based communication (e.g. Facebook Messenger, WhatsApp, chatrooms, SMS). What is important is that there was a continuous back‐and‐forth between you and the other person(s), and that this lasted for at least 2 minutes and was uninterrupted. For text‐based communication to count as an uninterrupted social exchange, there should not be a long gap (no more than a few minutes) between sending and receiving the messages. The exchange should feel similar to a conversation you may have with someone in person or on the phone, where there is a continuous and uninterrupted back‐and‐forth. If the exchange feels more like a correspondence you may have with someone via email, where there is usually a longer delay between receiving and sending the messages, then this should not count as an uninterrupted social exchange.

We defined a social interaction in this way to exclude fleeting social interactions, such as a brief greeting to a neighbour in the hallway or a single question‐and‐answer exchange via text or email (Lindsay et al., 2019). Participants were asked how many of the interactions since the previous data entry were in person (i.e., physically in the same location, i.e., ‘face‐to‐face’). If participants reported having at least one interaction since the last data entry, then they answered additional questions concerning their most recent interaction (response options are indicated in brackets after each question): *How many people took part in this exchange?* (Me plus one other person; more than two people); *Who was it with (tick all that apply)?* (partner; child; parents; grandparents; siblings; friend; colleague; stranger; other); *How close are you to this person? If the exchange was with more than two people*, *think about the person you are closest to in this group*. (0–100 visual analogue scale: not close at all‐very close); *How pleasant was this exchange?* (0–100 visual analogue scale: not pleasant at all –very pleasant); *How long was the exchange?* (less than 5; 5–20; 21–45; more than 45 min); *How did you communicate?* (in person [in the same physical place]; voice call [audio only]; video call; text‐based [Messenger, Chat]; other). At the end of each data entry, participants also indicated what activity they were currently engaged in (working, studying or engaging in free time) and each data entry was time‐stamped.

We carried out several adherence checks to ensure data quality. We removed self‐initiated evening entries completed after 06:00 (burst 1 = 74 entries; burst 2 = 27), entries which were started over 60 min after the prompt (burst 1 = 6; burst 2 = 3) and entries which took longer than 20 min to complete (burst 1 = 44; burst 2 = 35).

### Statistical analysis

We used the R package *lme4* (Bates et al., 2014) to conduct linear mixed‐effects models on our four dependent variables: stress, mood valence, calmness and energetic arousal. All models had two levels: level 1 contained the data from each data entry which was nested within participants on level 2. As recommended (Barr et al., 2013), we kept the random effects structure ‘maximal’ and thus included random intercepts and slopes for all predictor variables where possible (for the model output, data and analysis code for all models, see https://osf.io/5fude and also Supporting Information S2). Additionally, we included the corresponding mood or stress measure from the previous data entry as a covariate in all models (i.e., lag −1 serial autocorrelation; Linnemann et al., 2018). This ensured that the changes in stress or mood following data entries with a social interaction could not simply be attributed to levels of stress or mood *before* the interaction. For each preregistered hypothesis, we ran the following models:

- *H1 – momentary effects of social interactions*: to test the effect of a social interaction on each dependent variable, we coded the variable *social interaction* which indicated whether participants reported having engaged in at least one social interaction or had no interaction since the last data entry (1 = at least one interaction, 0 = no interaction).
- *H2* – *momentary effect of multiple interactions*: we created a variable *multiple interactions* which coded whether participants had just one interaction or more than one interaction since the last data entry (1 = more than one interaction, 0 = only one interaction) to determine whether more frequent interactions were related to changes in stress and mood.
- *H3 – the nature of the interaction*: to determine whether the medium of the interaction (face‐to‐face vs. not face‐to‐face, e.g., via audio or text), the valence of the interaction (i.e., how pleasant it was) and closeness of the interaction partner, influenced stress and mood, we ran a model for each stress and mood measure in which we included the variables *closeness*, *pleasantness* and *face‐to‐face interaction* in the same model. The variables *closeness* and *pleasantness* referred to participants' rating of how close they were to the interaction partner and the pleasantness of the interaction, respectively (see Methods). The variable *face‐to‐face interaction* coded whether the most recent interaction was face‐to‐face or not (1 = face‐to‐face interaction, 0 = an interaction which was not face‐to‐face, e.g., via video, audio or text). By including all three predictors in the model together, we could determine the unique contribution of each predictor over and above the effect of the others.
- *H4* ‐ *Sociability and the Prosocial Interactions*—to test whether the effect of social interactions on stress and mood would be modulated by how much people enjoy engaging in group interactions and having kind, reciprocal relationships as measured by the Sociability subscale and the Prosocial Interactions subscale of the SRQ, we included the interaction term between each of these subscales and the variable *social interaction* in each model.

In all analyses, we controlled for whether participants were engaged in free time at the time of the data entry (*free time*: 1 = free time, 0 = not free time) and the time of the data entry (*EMA time*). These two variables were included in all models as stress and mood fluctuate across the day and are strongly influenced by whether individuals are working or not (Feneberg Anja et al., 2022). *EMA time* was centred to 10:00 on each day, and all other level‐1 variables were participant‐mean‐centred (Enders & Tofighi, 2007). All level‐2 variables, that is, the SRQ subscales, were grand‐mean‐centred. Satterthwaite's method was used to test significance using *lmerTest* (Kuznetsova et al., 2017). Simple slopes analysis was used to explore interaction effects using *sim_slopes* from the package *jtools* (Long, 2022).

## RESULTS

### Descriptive statistics

On average, social interactions were relatively common with participants reporting at least one social interaction since the previous data entry in 72.63% (*SD* = 22.50%) of all data entries in burst 1 and 70.31% (*SD* = 23.21%) in burst 2. Participants reported at least one *in‐person* social interaction in 64.01% (*SD* = 29.04%) of all data entries in burst 1, and this figure increased in burst 2 to 68.57% (26.00%). Interactions tended to be with close others as mean closeness ratings (on a 0–100 scale) were 79.40 (*SD* = 14.63) in burst 1 and reduced to 73.75% (*SD* = 15.92) in burst 2. Three‐quarters of all reported interactions in burst 1 were with someone who was rated as 71.69 or higher on closeness, and this figure was 62.00 in burst 2. The mean pleasantness of the interactions did not change between burst 1 and burst 2 (see Table 1 for a direct comparison of burst 1 and burst 2). Results tables from the analyses are reported in Supporting Information S2.

### H1: Social interactions improved mood and reduced stress

In burst 1, data entries with at least one social interaction compared to those without a social interaction were associated with reduced momentary stress (estimate^1^ = − 0.883, *SE* = 0.433, *p* = .042) and increased mood valence (estimate = 2.605, *SE* = 0.371, *p* < .001), calmness (estimate = 1.579, *SE* = 0.381, *p* < .001) and energetic arousal (estimate = 3.014, *SE* = 0.440, *p* < .001). We replicated these effects in burst 2 for mood valence (estimate = 2.267, *SE* = 0.585, *p* < .001) and energetic arousal (estimate = 2.878, *SE* = 0.607, *p* < .001), but not for stress (*p* = .463) or calmness (*p* = .097). Recall that these changes in stress and mood following social interactions were not the result of difference in stress or mood *before* social interactions, as we included the corresponding stress or mood state in the data entry before the social interaction in all analyses.

### H2: More frequent social interactions improve momentary mood

Next, we determined whether having *more than one* social interaction compared with just one interaction since the previous data entry was related to changes in stress and mood by including the variable *multiple interactions* in each model. This analysis revealed that across both bursts having more than one interaction compared with just one interaction was related to greater momentary mood valence (burst 1: estimate = 1.202, *SE* = 0.367, *p* = .001; burst 2: estimate = 1.433, *SE* = 0.640, *p* = .026) and energetic arousal (burst 1: estimate = 2.382, *SE* = 0.431, *p* < .001; burst 2: estimate = 1.847, *SE* = 0.685, *p* = .007), but no significant change in stress (burst1: *p* = .162; burst 2: *p* = .924) or calmness (burst1: *p* = .431; burst 2: *p* = .591).

### H3: The nature of the interaction: face‐to‐face, pleasantness and closeness

In both burst 1 and burst 2, face‐to‐face interactions compared to those which were not face‐to‐face predicted reduced momentary stress (burst 1: estimate = −2.285, *SE* = 0.507, *p* < .001; burst 2: estimate = −2.059, *SE* = 0.978, *p* = .036), greater mood valence (burst 1: estimate = 1.759, *SE* = 0.412, *p* < .001; burst 2: estimate = 2.116, *SE* = 0.705, *p* = .003) and calmness (burst 1: estimate = 2.044, *SE* = 0.439, *p* < .001; burst 2: estimate = 1.772, *SE* = 0.760, *p* = .021), but no change in energetic arousal (burst 1: *p* = .488; burst 2: *p* = .389).

In both bursts, more pleasant interactions were associated with reduced stress (burst 1: estimate = −0.241, *SE* = 0.011, *p* < .001; burst 2: estimate = −0.253, *SE* = 0.021, *p* < .001), greater mood valence (burst 1: estimate = 0.311, *SE* = 0.009, *p* < .001; burst 2: estimate = 0.329, *SE* = 0.016, *p* < .001), calmness (burst 1: estimate = 0.274, *SE* = 0.010, *p* < .001; burst 2: estimate = 0.256, *SE* = 0.017, *p* < .001), and energetic arousal (burst 1: estimate = 0.130, *SE* = 0.012, *p* < .001; burst 2: estimate = 0.109, *SE* = 0.019, *p* < .001).

In both bursts, greater closeness of the interaction partner predicted lower mood valence (burst 1: estimate = −0.053, *SE* = 0.009, *p* < .001; burst 2: estimate = −0.062, *SE* = 0.013, *p* < .001), and calmness (burst 1: estimate = −0.032, *SE* = 0.009, *p* < .001; burst 2: estimate = −0.031, *SE* = 0.014, *p* = .023). In burst 1, greater closeness predicted greater stress (burst 1: estimate = 0.023, *SE* = 0.011, *p* = .029) and lower energetic arousal (burst 1: estimate = −0.030, *SE* = 0.011, *p* = .005) but not in burst 2 (stress: *p* = .545; energetic arousal: *p* = .172).

### H4: Sociability and the prosocial interactions

In burst 1, there was an interaction between the Prosocial Interactions subscale and the variable *social interaction* for mood valence (estimate = 1.422, *SE* = 0.718, *p* = .048), calmness (estimate = 2.013, *SE* = 0.736, *p* = .006) and energetic arousal (estimate = 1.703, *SE* = 0.853, *p* = .047), but not for stress (*p* = .253). Simple slopes analysis revealed that those participants scoring higher (+1*SD*) on the Prosocial Interactions subscale showed the greatest increase in mood valence in data entries in which they reported having had a social interaction (estimate = 3.346, *SE* = 0.529, *p* < .001) compared to those scoring lower (−1*SD*) on this subscale (estimate = 1.889, *SE* = 0.514, *p* < .001). The same pattern was seen for calmness and energetic arousal. Those scoring higher (+1*SD*) on the subscale showed an increase in calmness in data entries in which they reported having had a social interaction (estimate = 2.634, *SE* = 0.542, *p* < .001), but this increase was not seen in those scoring lower (−1*SD*) on this subscale (estimate = 0.572, *SE* = 0.527, *p* = .278). For energetic arousal, those scoring higher on the subscale (+1*SD*) showed a greater increase in energetic arousal in data entries with a social interaction (estimate = 3.912, *SE* = 0.626, *p* < .001) compared to those scoring lower (−1*SD*) on this subscale who showed a less marked increase (estimate = 2.167, *SE* = 0.609, *p* < .001).

There was a significant interaction between the Sociability subscale and the variable *social interaction* for mood valence (estimate = −0.577, *SE* = 0.284, *p* = .043), but not for the other dependent variables. Here, the interaction was in the opposite direction with those scoring higher (+1*SD*) on the Sociability subscale showing a less marked increase in mood valence (estimate = 1.842, *SE* = 0.528, *p* < .001) in data entries with a social interaction compared to those scoring lower (−1*SD*) on this subscale (estimate = 3.393, *SE* = 0.534, *p* < .001). In burst 2, no interaction term was significant for either subscale for any of the dependent variables (all *p*s > .05).

### Additional analyses

We also investigated the effect of momentary stress and mood on the probability of subsequent social exchanges (see Additional analyses in Supporting Information S2). We found that greater stress (burst 1: estimate = 0.003, *SE* = 0.001, *p* = .046), mood valence (burst 1: estimate = 0.003, *SE* = 0.002, *p* = .044) and energetic arousal (burst 1: estimate = 0.011, *SE* = 0.001, *p* < .001; burst 2: estimate = 0.009, *SE* = 0.002, *p* = .005) increased the probability of participants reporting a social interaction in the subsequent data entry. Moreover, we investigated whether social exchanges had an enduring effect by determining whether reporting a social exchange in the previous data entry predicted changes in stress and mood in the subsequent data entry (see Additional analyses in Supporting Information S1). We found that having social interaction in the previous data entry predicted lower stress (burst 1; estimate = −0.997, *SE* = 0.394, *p* = .012), greater mood valence (burst 1; estimate = 0.936, *SE* = 0.361, *p* = .010) and greater calmness (burst1; *p* = .009) suggesting an enduring beneficial effect of social interactions. However, in burst 2, we found that having a social interaction in the previous data entry predicted lower energetic arousal (burst 2: estimate = −1.643, *SE* = 0.621, *p* = .009) in the subsequent data entry.

## DISCUSSION

We conducted a preregistered EMA study during COVID‐19 lockdown in spring 2020 (burst 1: *n* = 732) and during a further lockdown in autumn/winter 2020 (burst 2: *n* = 281). Participants entered their momentary stress and mood into a smartphone app five times per day for 7 days. They also indicated whether they had engaged in social interaction since the last data entry and the nature of their most recent interaction, such as how pleasant it was, whether it was in person (i.e., face‐to‐face), and their closeness to their interaction partner. In burst 1, having had at least one social interaction since the previous data entry was associated with reduced stress and greater momentary mood valence, calmness and energetic arousal compared with entries in which there was no social interaction (H1). We replicated these effects in burst 2 for mood valence and energetic arousal. We also showed that more frequent social interactions in both burst 1 and 2 were associated with enhanced mood and energetic arousal thereby supporting H2. Importantly, these effects were found when controlling for mood or stress in the previous data entry and can thus not be explained by prior mood or stress. Our findings support studies showing that social contact during the COVID‐19 pandemic was associated with greater psychological well‐being (Nitschke et al., 2021; Sommerlad et al., 2021). By using an EMA approach—which reduces the impact of retrospective reporting biases—we complement and extend this previous work by demonstrating the *real‐time* affective benefits of social interactions during these psychologically demanding periods (Liu et al., 2019).

Our findings highlight the importance of face‐to‐face interactions for reducing momentary stress and improving mood during lockdown (H3). In both burst 1 and burst 2, face‐to‐face interactions, compared with interactions which were not face‐to‐face, were associated with reduced momentary stress, greater mood valence and calmness, even when controlling for the pleasantness of the interaction and the closeness of the interaction partner. Thus, face‐to‐face interactions were not simply more beneficial because they were more pleasant or with closer others (Sommerlad et al., 2021). Note that for all types of interactions (H1) we found large and consistent effects on mood but only a relatively small and marginal (*p* = .043) effect for reduced stress (and only in burst 1). Thus, face‐to‐face interactions may be particularly important for reducing stress as well as enhancing mood, whereas all types of social interactions may consistently boost mood but, on average, have weaker effects on reducing stress.

Previous EMA studies investigating the effects of different modes of communication have yielded mixed findings with some studies showing the benefits of digital communication (Gonzales, 2014) and others highlighting its inferiority compared with *in‐person* interactions (Dissing et al., 2019; Dunbar, 2012; Kross et al., 2013; Macdonald et al., 2021). Our data support studies showing a specific advantage for face‐to‐face interactions for psychological well‐being (Subrahmanyam et al., 2020), especially during the COVID‐19 pandemic (Sommerlad et al., 2021). The effects of face‐to‐face interactions during lockdown may have been particularly strong, as people had less frequent face‐to‐face interactions and the use of digital communication increased (Sun, Folarin, et al., 2020). In other words, face‐to‐face interactions may have been more of a ‘treat’ and had a greater impact on well‐being compared with pre‐lockdown. This shift to digital communication is likely to continue beyond the COVID‐19 pandemic as many individuals and organizations increasingly favour socializing (Twenge et al., 2019) and working (Phillips, 2020) remotely. Thus, it is important to determine whether the effects of face‐to‐face interactions on momentary stress and mood generalize beyond COVID‐19 lockdowns.

Interactions which were rated as more pleasant were associated with reduced stress and improved mood (H3), as previously shown (Bernstein et al., 2018). Importantly, we controlled for whether the interaction was face‐to‐face as well as the closeness of the interaction partner. Thus, this suggests that different types of interactions have the potential to improve affective states providing they are perceived as pleasant (Macdonald et al., 2021). One unexpected finding was that the greater closeness of the interaction partner was related to *reduced* mood valence and calmness in both lockdowns, and greater stress and lower energy in burst 1 (but not in burst 2). This contrasts with previous studies which have highlighted the importance of interacting with close others to boost happiness (Quoidbach et al., 2019). However, other studies have shown that, contrary to participants' expectations (Dunn et al., 2007), interactions with strangers can boost mood to the same extent as interactions with close others (Sandstrom & Dunn, 2014). Additionally, it is important to emphasize the unique context of lockdown, during which most interactions, especially in burst 1, were with close others (Table 1). Therefore, these differing effects of closeness on affective states following interactions could reflect differences between friends and family, rather than family and strangers. Furthermore, the content of conversations with closer others during the lockdown may have evoked stress and reductions in mood, although the interaction itself could have been pleasant.

In burst 1, we found that participants scoring higher on the Prosocial Interactions subscale of the Social Reward Questionnaire (SRQ; Foulkes et al., 2014) showed a greater increase in mood valence, calmness and energetic arousal in data entries with a social interaction compared to those scoring lower on this subscale (H4). The Prosocial Interactions subscale measures the extent to which participants enjoy having kind, reciprocal relationships (e.g., ‘I enjoy treating others fairly’). Across participants in burst 1, there was a correlation between the Prosocial Interactions subscale and both the mean pleasantness of the interaction and the mean closeness of the interaction partner (see Table S1). Therefore, the greater affective benefits of social interactions in individuals scoring higher on this subscale could be the result of these participants having more pleasant interactions and/or engaging in social interaction with closer others. Contrary to our predictions, participants in burst 1 scoring higher on the Sociability subscale of the SRQ showed an attenuated increase in mood valence in data entries with a social interaction compared to those scoring lower on this subscale (H4). This subscale measures the extent to which people enjoy engaging in group interactions (e.g., ‘I enjoy going to parties’). Sommerlad et al. (2021) showed that those people who reported being more socially active before the pandemic showed a stronger association between impaired social relationships and depressive symptoms. Our data build on these findings by highlighting that even when more sociable individuals engaged in social interactions during lockdown, they did not benefit from these to the same extent as less sociable individuals. This could explain why the participants in Sommerlad et al.'s study were particularly vulnerable to depressive symptoms during lockdown.

Our results cannot simply be explained by time of day, or the activity participants were engaged in during the time of the data entry (e.g., work or free time) as we controlled for these factors in all analyses. Furthermore, our additional analysis (see Supporting Information S1) demonstrated an enduring beneficial effect of social interactions for stress and mood. For example, in burst 1, social interaction in the previous data entry predicted lower stress and greater mood valence and calmness in the subsequent data entry.

Additionally, the effect of social interactions on stress and mood was not the result of participants having improved affect *before* the interaction as we included the corresponding affective state from the previous data entry as a predictor in all models. Moreover, in our additional analysis, we found participants in burst 1 were more likely to engage in *subsequent* social interactions when they reported greater stress, mood valence and greater energetic arousal in the previous data entry (though only the effect for energetic arousal was replicated in burst 2). Together, these findings fit with stress‐buffering accounts of social interactions (people seek social interactions to bring relief from aversive states; Cohen and Wills, 1985) but also with the view that people may seek social interactions to maintain or prolong positive affective states.

Our sample, especially in burst 1, was exceptionally large for an EMA study. However, it is important to highlight that we did not replicate all our findings across both lockdowns. For example, social interactions boosted mood in both lockdowns, but only reduced momentary stress in burst 1. Similarly, the SRQ subscales did not modulate the effect of social interaction on stress or mood in burst 2. These differing results could be the result of the reduced sample size and therefore lower power in burst 2, differences in the sample demographics (Table 1), and/or differences in the lockdown restrictions. That said, while future studies are needed to test the temporal robustness and generalizability of these effects, there was considerable consistency in the findings across the two lockdowns which were based on preregistered hypotheses.

A further limitation of our study was that we excluded participants who provided data in less than 50% of data entries. We preregistered this criterion to ensure sufficient power—our power calculation was based on a mean completion rate of 75% across participants. However, there are no clear guidelines or recommendations concerning the removal of non‐compliant participants within EMA research (Trull & Ebner‐Priemer, 2020). Indeed, the excluded participants in both bursts showed differences in their momentary stress and/or mood (see Tables S2 and S3) as well as in the frequency of social interactions (in burst 1). This raises the possibility that our findings only apply to those who showed reasonable compliance with the assessment protocol in what was already a fairly homogenous sample (see Table 1). Additionally, given that face‐to‐face interactions were severely restricted by the lockdown regulations, it is possible that there was some social desirability bias operating whereby participants underreported face‐to‐face social interactions.

Our results have potential implications for future lockdown policies. Firstly, given the clear benefits of face‐to‐face interactions, future lockdown restrictions must carefully weigh the relative risk of infection against the potential consequences for people's affective states, especially for those who live alone and do not have access to face‐to‐face interactions. Using a modelling approach, two studies both concluded that mixing of a single‐person household with another household during lockdown (thereby creating a social ‘bubble’) had a small impact on virus transmission (Danon et al., 2021; Leng et al., 2021). However, clearly policy makers must exercise caution as, when used ubiquitously, such bubbles could make transmission extremely hard to control (Danon et al., 2021). Secondly, our findings highlight that during lockdown not all individuals benefitted from social interactions to the same extent. Somewhat paradoxically more sociable individuals (as measured by the Sociability subscale of the SRQ) benefitted least from social interactions in terms of their momentary affect. This implies that these individuals may require extra support during psychologically demanding periods when their typical patterns of social contact are disrupted, thereby supporting previous findings (Sommerlad et al., 2021).

To conclude, social interactions, especially those which were face‐to‐face or rated as pleasant, were associated with reduced momentary stress and enhanced mood during COVID‐19 lockdowns—periods during which our social lives were severely disrupted. By demonstrating the real‐time, affective benefits of social interactions during lockdown, our results both validate and extend findings from cross‐sectional studies. Our data also highlight how certain individuals, such as those who enjoy having kind, reciprocal relationships, reap the affective benefits of social interactions to a greater extent. Conversely, individuals who reported being more sociable and engaging in group interactions benefitted from social interaction during lockdown to a lesser extent. These findings, by highlighting the importance of frequent and face‐to‐face social interactions for momentary affective states, have important implications for the (self‐)management of stress and mood during psychologically demanding periods.

## AUTHOR CONTRIBUTIONS

**Paul A G Forbes:** Conceptualization; data curation; formal analysis; investigation; methodology; writing – original draft; writing – review and editing. **Ekaterina Pronizius:** Investigation; methodology; software; writing – review and editing. **Anja C Feneberg:** Investigation; methodology; software; writing – review and editing. **Urs M Nater:** Funding acquisition; methodology; resources; supervision; writing – review and editing. **Giulio Piperno:** Investigation; methodology; writing – review and editing. **Giorgia Silani:** Funding acquisition; project administration; resources; writing – review and editing. **Ana Stijovic:** Data curation; investigation; methodology; software; writing – review and editing. **Claus Lamm:** Conceptualization; funding acquisition; investigation; methodology; project administration; resources; supervision; writing – review and editing.

## CONFLICT OF INTERESTS

The authors declare no conflict of interests.

## Supporting information


Supporting information S1.

Supporting information S2.

## Data Availability

The data and code for all the analyses are available on the Open Science Framework (see https://osf.io/5fude/).

## Endnote

^1^
Throughout the manuscript, ‘estimate’ refers to the unstandardized beta coefficient estimates.
