# Supplementary material for: The effects of social interactions on momentary stress and mood during COVID‐19 lockdowns
Source: Br J Health Psychol. 2022 Oct 17:10.1111/bjhp.12626. Online ahead of print. doi: 10.1111/bjhp.12626 (PMC9874800; doi:10.1111/bjhp.12626)
Supplement: Supplementary file 1 — Supporting information S1. [file BJHP-9999-0-s002.docx]

**Supplementary Materials**

**S1.** Overview of lockdown restrictions during burst 1 and burst 2

**Burst 1.** The first national lockdown began in Austria on March 16, 2020. People residing in Austria were only able to leave their homes for four reasons: for work (if this could not be done from home), to buy essential items (e.g., food, medicine), to carry out caring responsibilities, or for exercise (which could only be done alone or with those in one’s household). Lockdown restrictions began to be eased on May 1. For a detailed chronology of the pandemic in Austria, see <https://viecer.univie.ac.at/en/projects-and-cooperations/austrian-corona-panel-project/corona-blog/corona-blog-beitraege/blog51/>. In Italy, the first national lockdown began on 9 March, following a several regional lockdowns. As in Austria, people were only allowed to leave their homes for a limited number of reasons and non-essential businesses (e.g., bars, restaurants) were closed. Lockdown restrictions began to be eased on May 4 (see Bosa et al., 2021^[[1]](#footnote-1)^; for a timeline of COVID-19 lockdown restrictions in Italy). In Germany, national lockdown restrictions were implemented on March 22, 2020, although some regional restrictions had been in place before this. For a detailed chronology of the pandemic in Germany, see <https://www.dw.com/en/covid-how-germany-battles-the-pandemic-a-chronology/a-58026877>. Restrictions began to be eased on May 4.

**Burst 2.** In Austria, following a COVID-19 Emergency Action Order^[[2]](#footnote-2)^, a lockdown comparable to burst 1 was implemented on November 17, 2020 (until December 7, see <https://viecer.univie.ac.at/en/projects-and-cooperations/austrian-corona-panel-project/corona-blog/corona-blog-beitraege/blog100-en/>).

**S2.** Additional analyses

***The effect of momentary stress and mood on the probability of subsequent social exchanges***

We ran mixed effects logistic regression models with social interaction (1=yes, 0=no) as the dependent variable and the mood or stress measure from the previous data entry as the predictor (e.g., stress lagged) as well as the variables EMA time and free time. As in all analyses, we included these variables to control for time of day and the activity participants were engaged in at the time of the assessment.

For burst 1, we found that greater stress (estimate=0.003, SE=0.001, p=0.046), greater mood valence (estimate=0.003, SE=0.002, p=0.044) and energetic arousal (estimate=0.011, SE=0.001, p<0.001) increased the probability of participants reporting a social interaction in the subsequent data entry. However, calmness in the previous data entry did not predict the likelihood of a subsequent social interaction (p=0.250). For burst 2, we again found that greater energetic arousal in the previous data entry (estimate=0.009, SE=0.002, p=0.005) increased the likelihood of subsequent interaction. But stress (p=0.373), mood valence (p=0.282), or calmness (p=0.847) from the previous data entry did not predict the likelihood of a subsequent interaction.

These findings suggest that participants were more likely to engage in subsequent social interactions when they felt more stressed, reported greater mood valence or energetic arousal (although only the effect for energetic arousal was replicated in burst 2).

***Do social interactions have an enduring effect on momentary stress and mood?***

Next, we aimed to determine whether social interactions had an enduring effect on stress and mood by lagging the variable social interaction (*social lag*: this indicated whether participants had had a social interaction in the previous data entry; 1=yes, 0=no) and investigating its effect on stress and mood in the subsequent data entry. In each model, we included each mood or stress measure as the dependent variable and then included the variable *social lag* as a predictor variable. Additionally, we included the mood or stress measure from the previous data entry and, as in all models, the variables EMA time and free time.

For burst 1, having a social interaction in the previous data entry predicted lower stress (estimate=-0.997, SE=0.394, p=0.012), greater mood valence (estimate=0.936, SE=0.361, p=0.010), and calmness (estimate=0.918, SE=0.351, p=0.009) in the subsequent data entry suggesting an enduring beneficial effect of social interactions for stress and mood (although there was no change in energetic arousal; p=0.290). However, the effects from burst 1 were not replicated in burst 2 (stress: p=0.180; mood valence: p=0.873; calmness: p=0.539), instead having a social interaction in the previous data entry predicted lower energetic arousal in the subsequent data entry (estimate=-1.643, SE=0.621, p=0.009).

**Table S1**. Correlations coefficients between variables for burst 1 and 2. *Social interaction* refers to the proportion of completed data entries with a social interaction; *in person* refers to the proportion of completed data entries with an in person (i.e. face-to-face) social interaction; *Closeness* refers to the closeness of the social interaction partner; *Pleasantness* refers to the pleasantness of the social interaction, and *Sociability* and *Prosocial Interactions* refer to the subscales from the SRQ (Foulkes et al., 2014).

| **Burst 1** | % in person | Closeness | Pleasantness | Sociability | Prosocial Interactions |
| --- | --- | --- | --- | --- | --- |
| % social interactions | 0.37*** | 0.40*** | 0.23*** | 0.04 | 0.11** |
| % in person |  | 0.43*** | 0.14*** | -0.01 | 0.02 |
| Closeness |  |  | 0.49*** | 0.01 | 0.15*** |
| Pleasantness |  |  |  | -0.02 | 0.16*** |
| Sociability |  |  |  |  | 0.32*** |
| **Burst 2** | % in person | Closeness | Pleasantness | Sociability | Prosocial Interactions |
| % social interactions | 0.28*** | 0.39*** | 0.16** | 0.06 | 0.06 |
| % in person |  | 0.43*** | 0.11 | 0.05 | 0.03 |
| Closeness |  |  | 0.48*** | 0.15* | 0.11 |
| Pleasantness |  |  |  | 0.12* | 0.16** |
| Sociability |  |  |  |  | 0.29*** |

* p<0.5; **p<0.01,***p<0.001

**Table S2.** Comparison between the included and excluded participants for burst 1.

|  | **Included** | **Excluded** | ***P*** |
| --- | --- | --- | --- |
| N | 732 | 219 | - |
| Age | M=31.66 (SD=11.73) | M=30.89 (SD=12.15) | 0.409 |
| Gender | Female=516 (70.49%) | Female=146 (66.67%) | 0.280 |
| Stress | M=30.21 (SD=17.98) | M=32.97 (SD=18.09) | 0.048* |
| Mood valence | M= 63.31 (SD=14.71) | M=60.84 (SD=16.04) | 0.043* |
| Calmness | M=61.69 (SD=15.47) | M=58.51 (SD=16.57) | 0.012* |
| Energetic arousal | M=50.78 (SD=10.71) | M=48.90 (SD=12.18) | 0.041* |
| % social exchanges | M=72.63% (SD=22.50) | M=71.92 (SD=26.63**)** | 0.723 |
| % face-to-face exchanges | M=64.01% (SD=29.04) | M=67.82 (SD=31.37) | 0.113 |

**Table S3.** Comparison between the included and excluded participants for burst 2.

|  | **Included** | **Excluded** | ***P*** |
| --- | --- | --- | --- |
| N | 281 | 76 | - |
| Age | M=34.18 (SD=13.16) | M=31.83 (SD=11.30) | 0.123 |
| Gender | Female=221 (78.65%) | Female=59 (77.63%) | 0.848 |
| Stress | M=28.20 (SD=18.20) | M=27.69 (SD=16.40) | 0.817 |
| Mood valence | M=63.60 (SD=14.71) | M=61.07 (SD=13.97) | 0.170 |
| Calmness | M=61.14 (SD=15.89) | M=58.91 (SD=14.51) | 0.247 |
| Energetic arousal | M=47.98 (SD=11.28) | M=44.75 (SD=10.50) | 0.021* |
| % social exchanges | M=70.31% (SD=23.21) | M=75.93% (SD=19.55) | 0.035* |
| % face-to-face exchanges | M=68.57% (SD=26.00) | M=77.48% (SD=25.64) | 0.008** |

1. Bosa I, Castelli A, Castelli M, et al. Response to COVID-19: was Italy (un)prepared? Health Economics, Policy and Law. 2021:1-13. doi:10.1017/S1744133121000141 [↑](#footnote-ref-1)
2. <https://www.ris.bka.gv.at/Dokumente/BgblAuth/BGBLA_2020_II_479/BGBLA_2020_II_479.html> [↑](#footnote-ref-2)
