# Supplementary material for: The effects of social interactions on momentary stress and mood during COVID‐19 lockdowns
Source: Br J Health Psychol. 2022 Oct 17:10.1111/bjhp.12626. Online ahead of print. doi: 10.1111/bjhp.12626 (PMC9874800; doi:10.1111/bjhp.12626)

**Output from all models**

The tables below show the results of all the models for each hypothesis and each measurement burst. Estimate = unstandardized coefficient. CI = 95% confidence interval. The prefix ‘pmc’ indicates that the variable was participant mean centered and the prefix ‘gmc’ indicates that it was grand mean centered. The suffix ‘Lag’ indicates that this was the corresponding mood or stress measure from the previous data entry. The tables were create using the *tab_model* function from the R package *sjPlot* (Lüdecke, 2021).

**Hypothesis 1: Burst 1**


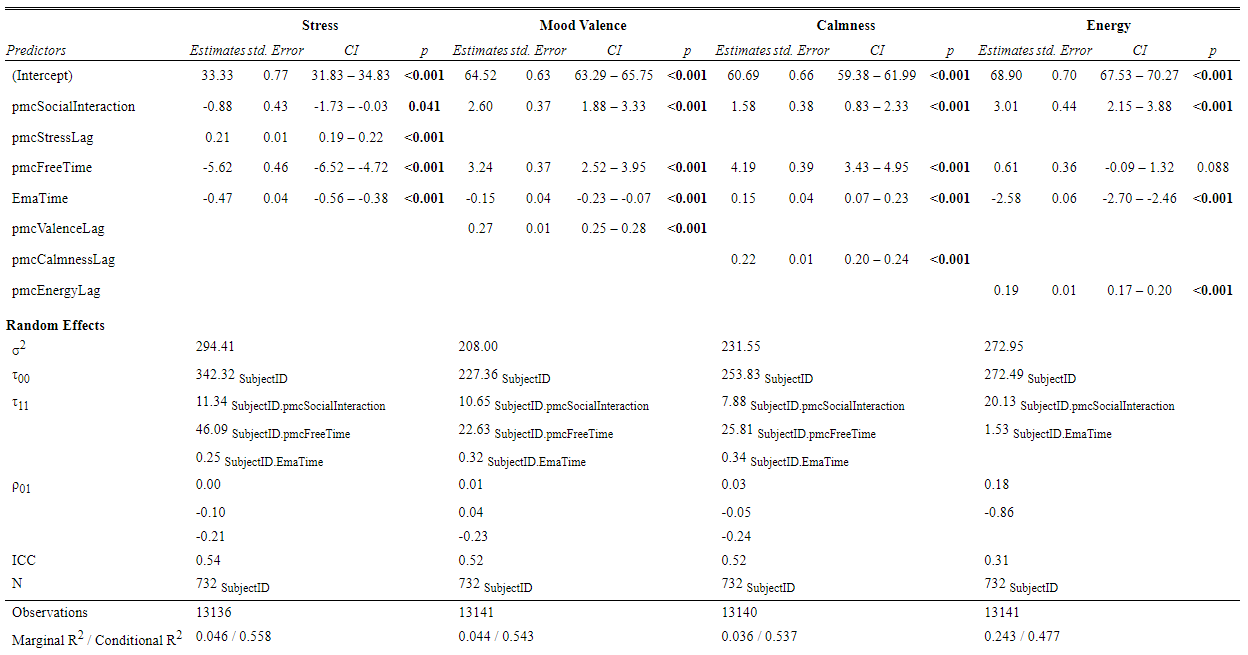


**
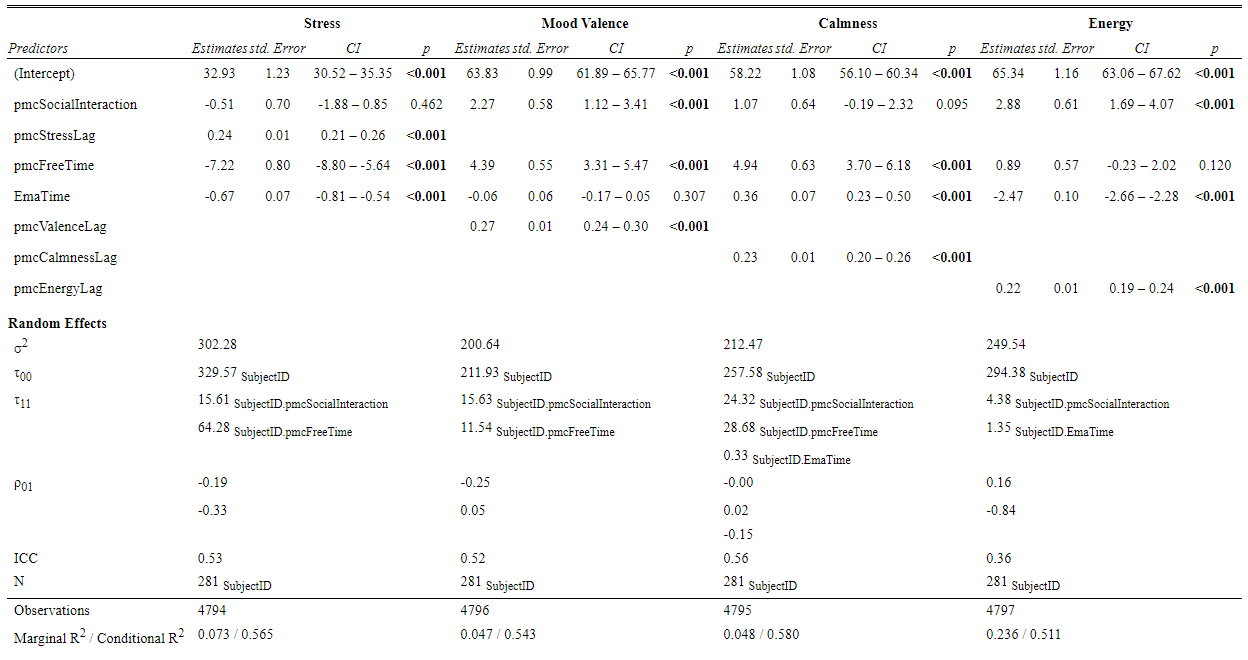
Hypothesis 1: Burst 2Hypothesis 2: Burst 1**
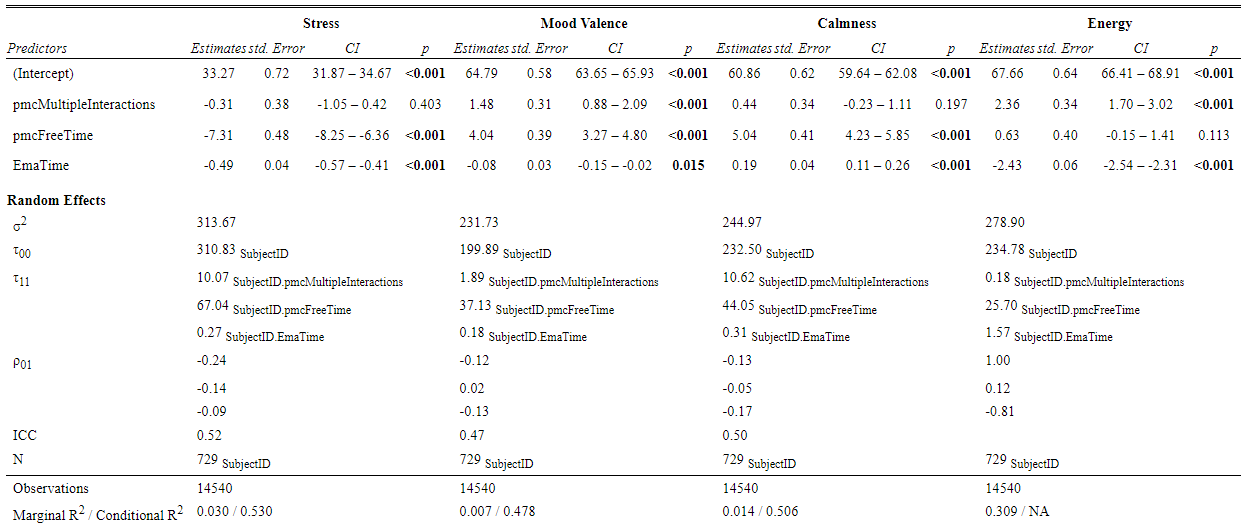


**Hypothesis 2: Burst 2**
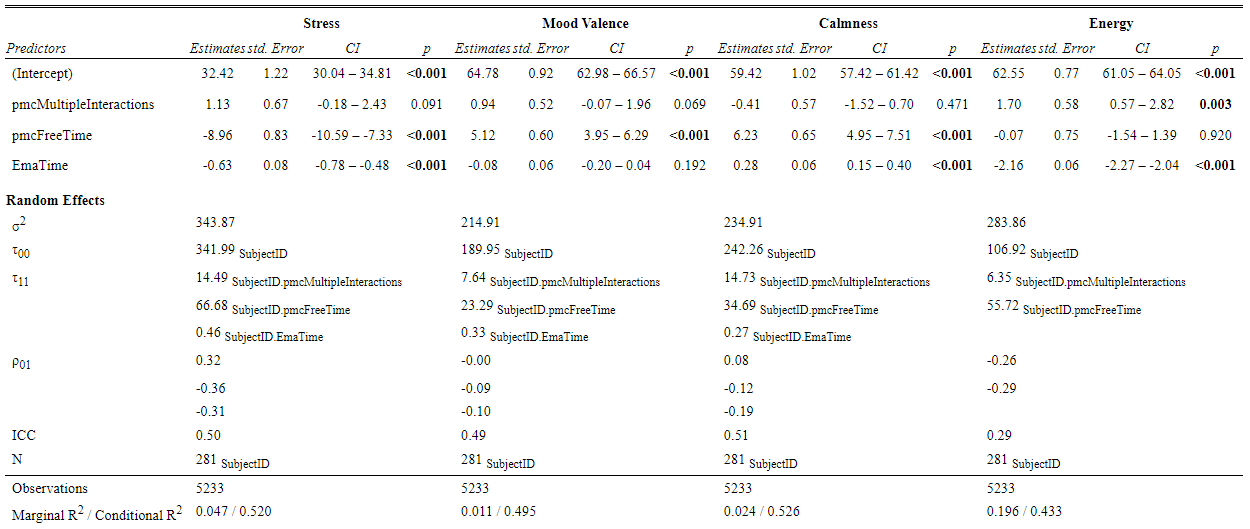


**Hypothesis 3:** **Burst 1**


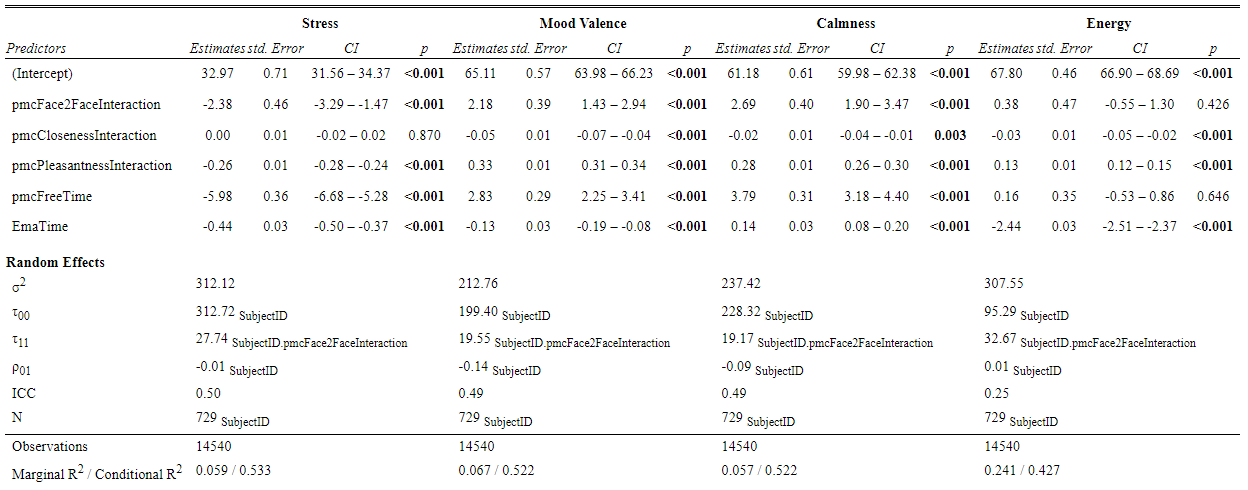


**Hypothesis 3: Burst 2**


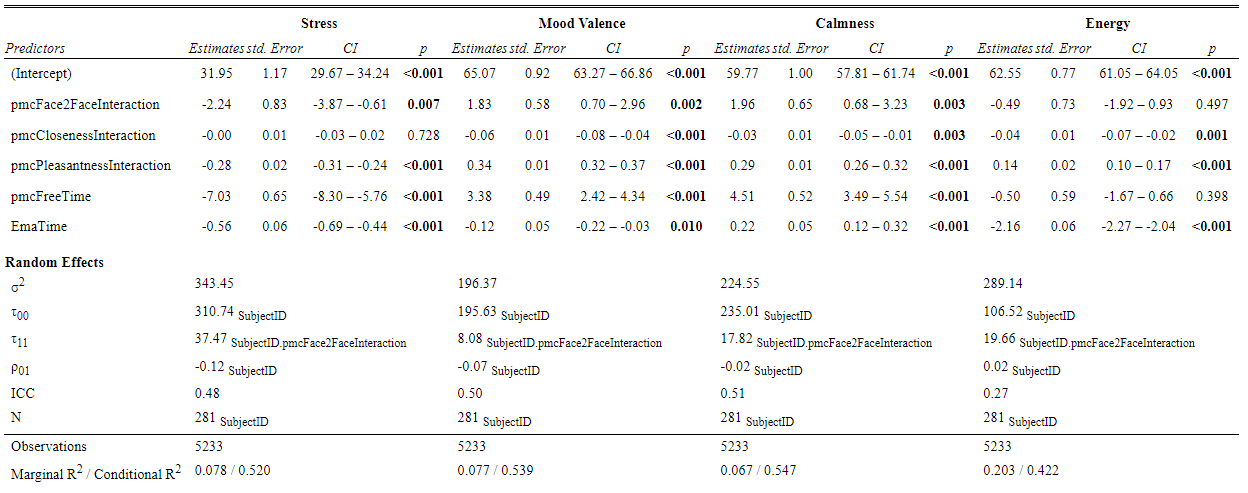


**Hypothesis 4: Burst 1**
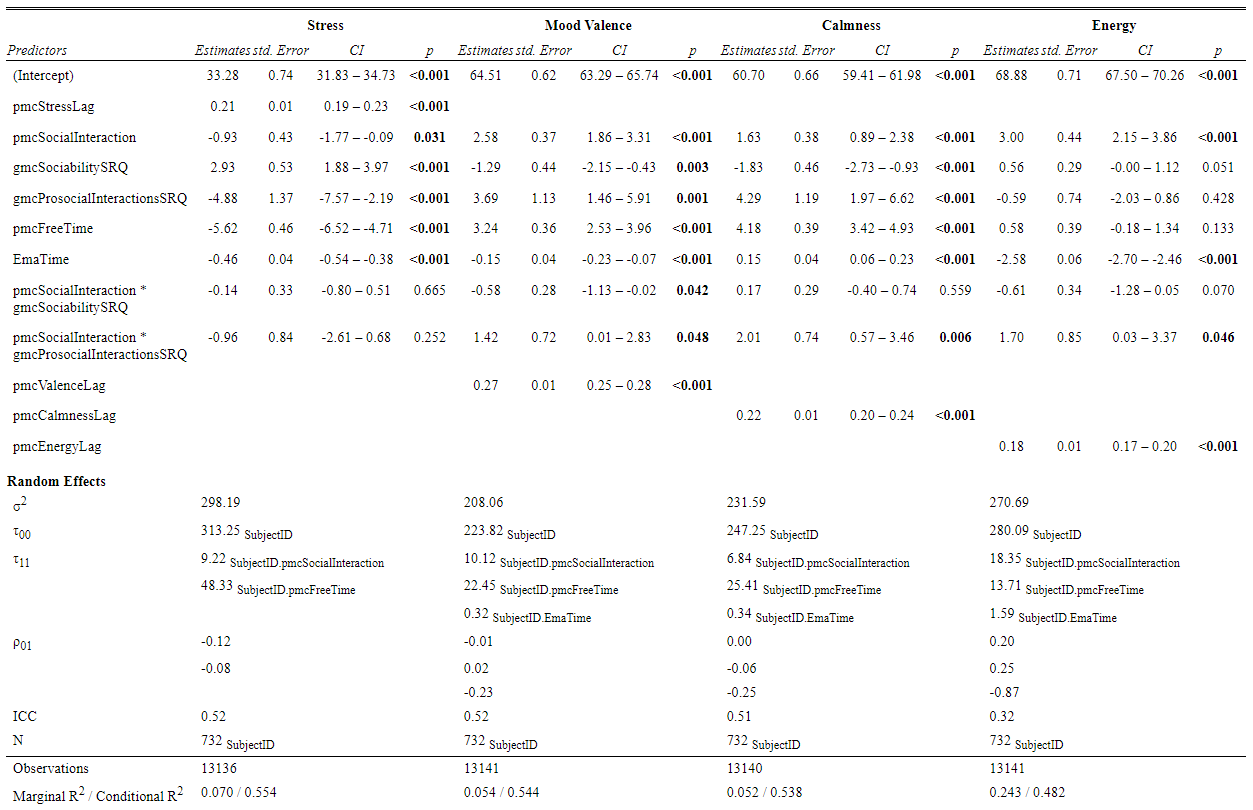


**Hypothesis 4: Burst 2**
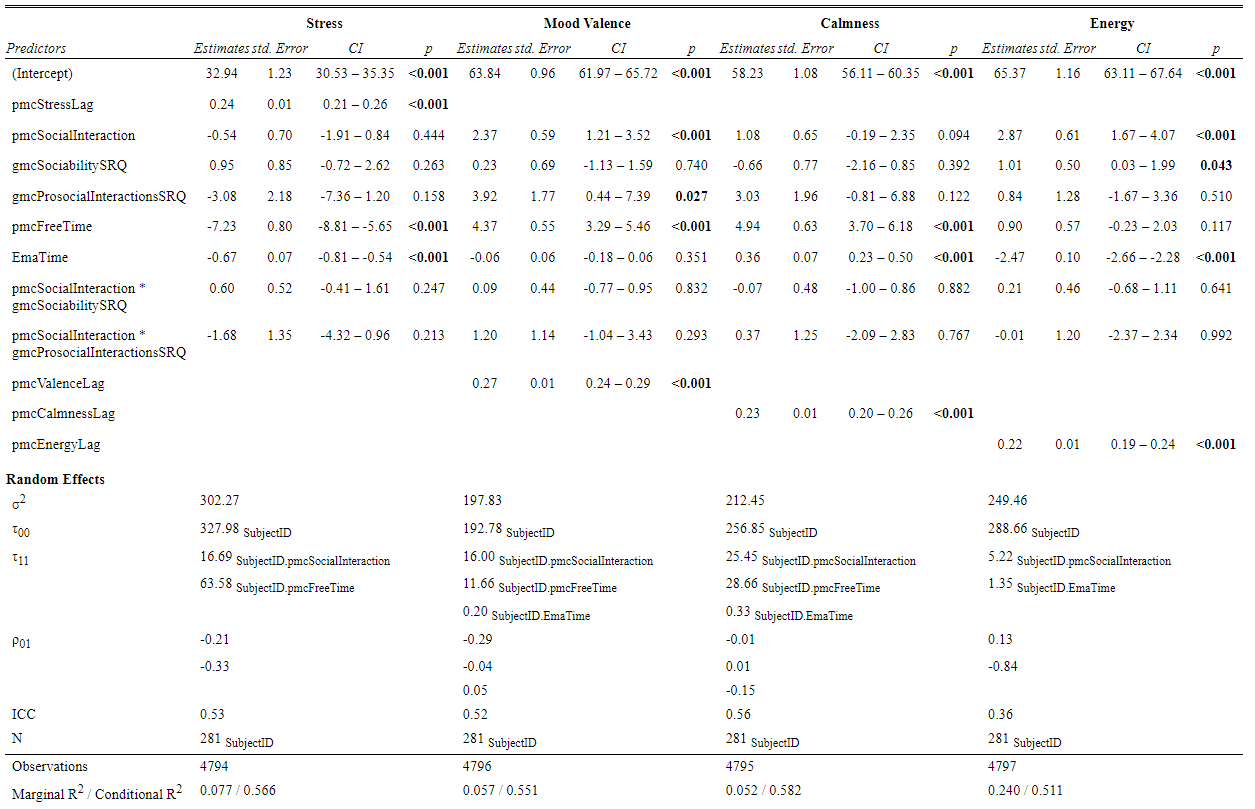

Supplement: Supplementary file 2 — Supporting information S2. [file BJHP-9999-0-s001.docx]
